# Preclinical evidence for anaplastic lymphoma kinase inhibitors as novel therapeutic treatments for cholangiocarcinoma

**DOI:** 10.3389/fonc.2023.1184900

**Published:** 2023-12-07

**Authors:** Kyaw Zwar Myint, Mireia Sueca-Comes, Pamela Collier, Brinda Balasubramanian, Simran Venkatraman, John Gordan, Abed M. Zaitoun, Abhik Mukherjee, Arvind Arora, Noppadol Larbcharoensub, Chinnawut Suriyonplengsaeng, Kanokpan Wongprasert, Tavan Janvilisri, Dhanny Gomez, Anna M. Grabowska, Rutaiwan Tohtong, David O. Bates, Kiren Yacqub-Usman

**Affiliations:** ^1^Graduate Program in Molecular Medicine, Faculty of Science, Mahidol University, Bangkok, Thailand; ^2^Division of Cancer and Stem Cells, Biodiscovery Institute, School of Medicine, Biodiscovery Institute, University of Nottingham, Nottingham, United Kingdom; ^3^Department of Medicine, University of California, San Francisco, San Francisco, CA, United States; ^4^Department of Pathology, Nottingham Universities National Health Service (NHS) Hospital Trust, Queens Medical Centre, Nottingham, United Kingdom; ^5^Department of Medical Oncology, Nottingham Universities National Health Service (NHS) Hospital Trust, Queens Medical Centre, Nottingham, United Kingdom; ^6^Department of Pathology, Faculty of Medicine Ramathibodi Hospital, Mahidol University, Bangkok, Thailand; ^7^Department of Anatomy, Faculty of Science, Mahidol University, Bangkok, Thailand; ^8^Department of Biochemistry, Faculty of Science, Mahidol University, Bangkok, Thailand; ^9^Department of Hepatobiliary and Pancreatic Surgery, and National Institute of Health Care Research (NIHR) Nottingham Digestive Disease Biomedical Research Unit, University of Nottingham, Nottingham, United Kingdom

**Keywords:** cholangiocarcinoma, *Opisthorchis viverrini*, anaplastic lymphoma kinase 1, novel therapeutics, cytotoxicity and autophagy

## Abstract

**Introduction:**

Bile duct cancer (cholangiocarcinoma, CCA) has a poor prognosis for patients, and despite recent advances in targeted therapies for other cancer types, it is still treated with standard chemotherapy. Anaplastic lymphoma kinase (ALK) has been shown to be a primary driver of disease progression in lung cancer, and ALK inhibitors are effective therapeutics in aberrant ALK-expressing tumors. Aberrant ALK expression has been documented in CCA, but the use of ALK inhibitors has not been investigated. Using CCA cell lines and close-to-patient primary cholangiocarcinoma cells, we investigated the potential for ALK inhibitors in CCA.

**Methods:**

ALK, cMET, and ROS1 expression was determined in CCA patient tissue by immunohistochemistry and digital droplet polymerase chain reaction, and that in cell lines was determined by immunoblot and immunofluorescence. The effect on cell viability and mechanism of action of ALK, cMet, and ROS1 inhibitors was determined in CCA cell lines. To determine whether ceritinib could affect primary CCA cells, tissue was taken from four patients with biliary tract cancer, without ALK rearrangement, mutation, or overexpression, and grown in three-dimensional tumor growth assays in the presence or absence of humanized mesenchymal cells.

**Results:**

ALK and cMet but not ROS were both upregulated in CCA tissues and cell lines. Cell survival was inhibited by crizotinib, a c-met/ALK/ROS inhibitor. To determine the mechanism of this effect, we tested c-Met-specific and ALK/ROS-specific inhibitors, capmatinib and ceritinib, respectively. Whereas capmatinib did not affect cell survival, ceritinib dose-dependently inhibited survival in all cell lines, with IC_50_ ranging from 1 to 9 µM and co-treatments with gemcitabine and cisplatin further sensitized cells, with IC_50_ ranging from IC_50_ 0.60 to 2.32 µM. Ceritinib did not inhibit cMet phosphorylation but did inhibit ALK phosphorylation. ALK was not mutated in any of these cell lines. Only ceritinib inhibited 3D growth of all four patient samples below mean peak serum concentration, in the presence and absence of mesenchymal cells, whereas crizotinib and capmatinib failed to do this. Ceritinib appeared to exert its effect more through autophagy than apoptosis.

**Discussion:**

These results indicate that ceritinib or other ALK/ROS inhibitors could be therapeutically useful in cholangiocarcinoma even in the absence of aberrant ALK/ROS1 expression.

## Introduction

Our understanding of the pathogenic mechanisms responsible for the evolution and outgrowth of cholangiocarcinomas (CCA) is far from complete. Moreover, this heterogenous cancer consists of a milieu of transcription factors, growth factors, and their cognate receptors that are molecularly heterogenous and subtype-specific and differ dependent on their geographical location. The incidence and mortality rates are increased in the Mekong basin areas of Southeast Asia (Thailand, Loas, and Vietnam) due to infection with the liver fluke *Opisthorchis viverrini* (Ov-CCA) compared with CCA not associated with Ov (Non-Ov CCA). Current treatments include surgical resection with an overall median survival rate of 2 years and standard systemic treatment of combination of gemcitabine-based therapies in combination with cisplatin, oxaliplatin, capecitabine, or 5-FU (1), resulting in very small increases in overall survival. Recent characterization studies have identified recurrent genetic alterations in CCA, which may be amenable to therapeutic targeting.

Studies from several groups have explored the potential of overexpression of cMet and preclinical characterization of novel inhibitors of the cMet pathway as treatment options for cholangiocarcinomas. In CCA cell lines and *in vivo* studies, the selective cMet inhibitor capmatinib inhibited cMet phosphorylation and activation of key downstream effectors of cMet, and as a result inhibits cell proliferation and migration and induces apoptosis (2). MET-amplified (MKN45) and MET-overexpressed (H441) xenograft models of CCA were used to further the preclinical development of an orally bioavailable small-molecule inhibitor LY2801653 targeting MET kinase leading to *in vivo* anti-tumor effects and *in vivo* vessel normalization effects (3). LY2801653 is currently in phase 1 clinical trials in patients with advanced disease (NCT01285037). Indeed, several subsequent clinical trials are currently being performed in CCA, but all have limitations due to underpowered studies, mixed cohorts, and design without intent to enrich for markers, required to optimize success for targeted therapies (4, 5).

Anaplastic lymphoma kinase (ALK) (6), hepatocyte growth factor receptor (HGFR/c-Met) (7), and ROS proto-oncogene 1 receptor tyrosine kinase (ROS1) (8) are tyrosine receptor kinases, implicated to be aberrantly expressed in human cancers (9–11). ALK, c-Met, and ROS1 were reported to have high expression in advanced biliary tract carcinoma and patients with high expression of all 3 proteins by IHC staining had a significantly inferior median overall survival than patients with low-expressing tumors (12). CCA tissues were reported to have high c-Met expression; however, c-Met expression in CCA was reported with contradicting findings on disease-free survival and treatment response to standard gemcitabine and cisplatin therapy (12–14).

In addition, subsequent investigations into multi-targeted agents to treat CCA have been employed. Crizotinib is a multi-targeted tyrosine kinase inhibitor, targeting ALK, c-Met, and ROS1, and FDA approved for the treatment of non-small cell lung cancer (NSCLC) patients with ALK rearranged mutations and melanoma patients (15–17). These studies showed an objective response rate of 72% with a median progression-free survival of 19.2 months of NSCLC patients treated with crizotinib with high ROS1 levels and resulted in further therapeutic options of multi-targeted treatment for CCA (18). Furthermore, ceritinib (LDK378), a more potent second-generation tyrosine kinase inhibitor with higher anaplastic lymphoma kinase (ALK) selectivity than crizotinib (19), has been investigated and shown to be active against NSCLC. ALK overexpression is found in several cancers included but not limited to ovarian, progressive neuroblastoma, lung cancer, and cholangiocarcinoma.

The purpose of this study was to gain further insight into the relationship and interplay between the expression of ALK, cMet, and ROS1 and the cytotoxicity of crizotinib, ceritinib, and capmatinib in cholangiocarcinoma cells. We therefore determined the expression of ALK, cMet, and ROS in surgically resected patient tissue, primary cell lines derived from surgically resected patient tissue, and immortalized cell lines. In addition, we determined the cytotoxicity of crizotinib, ceritinib, and capmatinib. Furthermore, we also used a knockdown strategy towards ALK, cMet, and ROS1 to determine the effect on cell proliferation. We investigated the possible mechanism of action of these inhibitors in CCA. The efficacy of each inhibitor was further confirmed using primary cells derived from surgically resected patient tissue in a 3D tumor growth assay and compared to serum plasma concentrations. This work shows for the first time that ceritinib is a potential therapeutic option for the treatment of cholangiocarcinoma.

## Materials and methods

### Ethics approval and patient consent statement

Surgical material from tumor resections at Nottingham University NHS trust were collected with informed patient consent and National Research Ethics Service approval (NRES REC 10/H0405/6). Samples were used in accordance with NRES approval (NRES REC 08/H0403/37). The study protocol in Thailand was approved by the ethical clearance committee on human rights related to research involving human subjects, Faculty of Medicine Ramathibodi Hospital, Mahidol University (protocol no. 12-58-41) and Rajavithi Hospital (protocol no. 61042).

### Specimen collection

Fresh surgical material from tumor resections at Nottingham University NHS trust were collected with informed patient consent and National Research Ethics Service approval (NRES REC 10/H0405/6). Samples were used in accordance with NRES approval (NRES REC 08/H0403/37). Surgical material was collected and processed as previously described (20). In brief, surgical material was immediately placed into tissue transfer and transferred to the laboratory and processed within 4–6 h.

Samples were dissected upon arrival at the laboratory. The majority of tissue was used for live use, further portions were snap frozen for protein analysis, formalin-fixed and paraffin-embedded (FFPE) for immunohistochemistry, or stored in RNAlater (Ambion) for subsequent analysis. A small amount of finely minced tumor tissue was enzymatically disaggregated [as described below (20)].

### Disaggregation of tissue

Finely minced tumor was disaggregated using type II collagenase (100 U/mL: Invitrogen) and dispase (2.4 U/mL; Invitrogen) in Hanks’ balanced salt solution (HBSS) without calcium or magnesium (Sigma, UK) at 37°C under constant rotation. Cells were removed at hourly intervals until the tumor was completely disaggregated. Cell number and viability were determined using trypan blue exclusion as previously described.

### Establishing close-to-patient cells using a feeder layer method

*In vitro* tumor cell growth was established and expanded with a layer of supporting feeder layer cells according to the method of Liu et al. (21) and as previously described (22). In brief, from this material, the tumor epithelial cells were expanded and harvested separately from the cancer-associated fibroblasts using differential trypsinization. Tumor cell number and viability were determined using trypan blue exclusion. At less than passage 5, cell aliquots were cryopreserved, utilized for 3D-TGA and further experimentation. Primary cells used within this study were as follows: CCA-UK5: extrahepatic adenocarcinoma, CCA-UK6: well-differentiated intrahepatic carcinoma, CCA-UK7: multi-desmoplastic and poorly differentiated perihilar, and CCA-UK9: extrahepatic adenocarcinoma low in stroma and epithelial alignments.

### The 3D-tumor growth assay

As previously described (20), in brief, cells were resuspended in ice-cold Cultrex basement membrane extract (BME) (9 mg/mL: Trevigen) diluted in modified RPMI-1640 (life technologies; phenol red free with 6 mmol/L D-Glucose and pH 6.8) and plated at 6,250 tumor cells ± mesenchymal cells (bone marrow derived) (ScienCell) into low adherent, black-walled, clear-bottom, 384- well plates. Drugs were serially diluted in modified RPMI-1640 and 13 μL of drug was added in six replicates on day 3. Drugs used in combination were premixed and serially diluted together before adding to the assay. Drug exposure was for 96 h before final endpoint readings. The AlamarBlue assay [Invitrogen; 10% (v/v), 37°C for 1 h] was used to monitor cell growth daily using fluorescent plate reader (FLUOstar Omega, BMG Labtech). Drug sensitivity was calculated as a percentage of matched untreated control and IC_50_ curves were determined using GraphPad Prism 5 (GraphPad Software Inc, nonlinear curve fit of Y = 100/(1 + 10 ^((Log^_10_^IC^_50_^-X)^*HillSlope). Error bars represent one standard error of the mean. Drugs in combination were at constant ratios to make them amenable to synergy testing using Chou-Tala-Lay method and CalcuSyn software.

### Cell cultures

RBE, a human intrahepatic cholangiocarcinoma cell line, TFK-1, a human extrahepatic cholangiocarcinoma cell line, KKU-M213 and KKU-M156, two human intrahepatic cholangiocarcinoma cell lines from a patient with *Opisthorchis viverrinae* infection, and KKU-M055 from a different patient with intrahepatic cholangiocarcinoma cell line and *Opisthorchis viverrinae* infection and HuCCA-1, a human intrahepatic cholangiocarcinoma, were all obtained from Professor John Gordan, University of California under a material transfer agreement. All cell lines were cultured in DMEM supplemented with 10% fetal bovine serum and 2 mM L-glutamine (Sigma-Aldrich, UK) in 5% _CO2_ atmosphere at 37°C. Cells in the current study had undergone 10 passages.

### Inhibitors

Inhibitors employed within these studies were crizotinib, ceritinib, capmatinib, and bafilomycin. Inhibitors used at the University of Nottingham were commercially bought from Selleckchem (Munchen, Deutschland), and inhibitors used at Mahidol University, Thailand were kindly provided by Assistant Professor Dr. Pimtip Sanvarinda, Department of Pharmacology, Faculty of Science, Mahidol University, Thailand. The drugs were dissolved in cell culture grade dimethyl sulfoxide (DMSO) (AppliChem, Barcelona, Spain) to prepare 5–30 mM stocks. Frozen DMSO stocks were prepared together with the drug to be used as vehicle. All drug treatments were performed at 60%–80% cell confluence. Hepatocyte growth factor (HGF) was purchased from Immunotools, Friesoythe, Germany and 100 µg/µl stock were prepared in 0.1% BSA/PBS.

### Expression analysis

#### Absolute expression analysis (digital droplet reverse transcriptase PCR)

RNA was extracted and purified from surgically resected tumors, five primary cell lines (CCA-UK5, CCA-UK6, CCA-UK7, CCA-UK9, and CCA-UK11), and patient matched, histologically tumor free resected margin as a control as previously described. Total RNA (1 μg) was reverse transcribed using 500 ng of Oligo -d(T) and 250 ng of random primers and M-MLV reverse transcriptase (Takara) in 20 µl total volume. cDNA (1 μL) was added to 1 μL of Taqman Probe and 8 μL of ddH_2_O with10 μL of ddPCR supermix for probes (Bio-Rad, UK). Droplets were generated using QX100 droplet generator (Bio-Rad, UK). Amplification was carried out using standard Taqman protocols with probe found in **Table 1.0** in **Supplementary Information**). Samples were analyzed using QX100 droplet reader (Bio-Rad, UK). Thresholding was manually performed, based on negative and positive control results only when automatic thresholding of values was not possible. Data are expressed as copies of RNA per µg of total RNA.

### RT-qPCR

qRT-PCR was performed using Faststart universal SYBR green Master (Roche, Mannheim, Germany). Primer sequence to ALK gene (NM_004304.5): forward: 5’-GAGGGGGCGGCAAGATT-3’ and reverse: 5’-CTTGTGGCTCCTCCAAGCTC-3’ were employed. The target gene was normalized to an endogenous control, 18S mRNA, forward: 5’-CCATCCAATCGGTAGTAGCG-3’ and reverse: 5’ GTAACCCGTTGAACCCCATT-3’. Relative quantification was carried out using relative-standard curve and the 2^−ΔΔCT^ method, where −ΔΔCT = cycle threshold (CT) (ALK of treated cell lines − 10S of treated cell line) − CT (ALK control − 18S control).

### Protein expression analysis

Total protein was extracted using NP-40 lysis buffer containing 1% (vol/vol) Triton X (Calbiochem, Germany), 1 × Protease Inhibitor (Roche, Germany), 50 mM NaF (Sigma, UK), 2 mM sodium orthovanadate (Sigma, UK), and phenylmethylsulfonyl fluoride (PMSF) (Sigma, UK) and quantified using Pierce® BCA Assay Kit (Thermo Scientific, UK); 25–40 μg (UK and Thailand respectively) were separated via SDS-PAGE on 12% gels followed by dry transfer of the proteins to nitrocellulose membrane using the Trans-Blot Turbo Transfer System (Bio-Rad, UK) for 10 min. The membranes were blocked in 5% BSA for 1.5 h. Blots were then incubated with primary antibodies (**Table 2.0** in **Supplementary Information**) overnight. The blots were then incubated with IRDye fluorescent secondary antibodies for 1 h at room temperature and imaged using the LiCOR Odyssey for imaging. All blots were analyzed and quantified using Image Studio™ (LI-COR®).

### Immunohistochemistry

Immunohistochemistry was used to examine the expression of ALK, cMET, and ROS1 receptors in CCA patient tissues from tumors with different etiologies (OV related in the Thai group and non-Ov in the UK group) and previously described. FFPE tumor samples were retrieved from the Department of Pathology, Ramathibodi Hospital, Mahidol University and Department of Pathology, Rajavithi Hospital, Bangkok, Thailand and also patients from the Department of Pathology, Nottingham Universities Hospital Trust, for immunohistochemistry assessment. All tissues were confirmed as mass forming CCA by the two pathologists of each institute (NL and CS, and AZ and AM). OV-related CCA in Thai patients was diagnosed by the patients fulfilling one of the five criteria as previously described (20). Each case was tested using the following primary antibodies: anti-cMET (#3077, Cell Signalling, UK), anti-ALK (ab190934, abcam, UK), and anti-ROS1(#3287, Cell Signalling, UK) antibodies. Serial 5-μm sections of FFPE tissue were sectioned by microtome for immunohistochemical analysis. The immunoreactivity was scored based on membrane and or cytoplasmic staining compared to positive controls as follows: no staining or faint staining < 10% of tumor cells; 1+, weak perceptible membranous staining in >10% of tumors; 2+, moderate complete membranous staining in >10% of tumor cells; 3+, strong complete membranous staining in >10% tumor cells (23).

### Cell viability and proliferation assay

Cell proliferation and viability were determined by WST-1 assay (Sigma, UK). The cells were seeded in 96-well plate at a density of 5,000 cells per well, and 24 h post seeding, the cells were treated with 0.1, 1, 10, and 100 μM of crizotinib, ceritinib, or capmatinib. The treatments and drug combinations are shown in figure legends. Cells were incubated with drugs for 48 h and cell proliferation was determined, WST-1 assay was added 10% (v/v) and incubated for 45 min, and absorbance was measured 450 nm by a plate reader. Cells in autophagy reversal experiments were pre-treated with bafilomycin (10 nM) and then subjected to the IC_50_ dose of ceritinib, crizotinib, or capmatinib at 10 µM (IC_50_ not reached).

### Receptor stimulation assay

Receptor stimulation was performed by initially blocking the receptor with specific inhibitor prior to stimulation of the receptor with receptor specific ligand. Cholangiocarcinoma cell lines were seeded at a density of 200,000 cells per well in a six-well plate for 24 h. The cells were serum starved in 0% FBS medium for a further 24 h. Cells were treated with 5 µM of either crizotinib, ceritinib, or capmatinib for 2 h and then stimulated with HGF 40 ng/mL added to the inhibitor containing medium for 1 h and protein extraction was performed.

### siRNA-mediated gene silencing

siRNA targeting ALK genes, siALK-1 5′-GAGUCUGGCAGUUGACUUCTT-3′ targeting Exon-1 of ALK mRNA (24) and siALK-2 5′-GUGCCAUGCUGCCAGUUAAUU-3′ targeting Exon-26 kinase region of ALK mRNA (25), cMET gene, ROS1 gene, and a non-targeting siRNA (siNT) were synthesized commercially (Eurofins Genomics, Ebersberg, Germany). The transfection complex siRNA and RNAiMAX (Life technologies, UK) were prepared. RBE, TFK-1, KKU-M055, KKU-M156, and KKU-M213 cell lines were reversed transfected with the transfection complex. Low passage cells at a density of 200,000 cells were transfected with 15 nM siRNA or the non-target control according to the manufacturer’s instructions (Life Technologies, Paisley UK). Cells were transfected for 48 h prior to RT-qPCR and apoptosis assays. Experiments were carried out thrice independently.

### Apoptosis assay

Apoptosis was determined using the Caspase 3/7 Glo Assay (Promega, UK) as per the manufacturer’s instructions. The cells were seeded in a 96-well plate at a density of 5,000 cells per well, and 24 h post seeding, the cells were treated with 0.1, 1, 10, and 100 μM of crizotinib, ceritinib, or capmatinib. The treatments and drug combinations are shown in figure legends. Cells were incubated with drugs for 48 h and caspase activity was determined, Caspase 3/7 Glo assay was added 10% (v/v) and incubated for 45 min and luminescence was measured by a plate reader.

### Statistical analysis

All experiments were repeated at least three times and presented as the mean ± standard error of the mean (SEM) of three independent experiments. Comparison of data between two groups was performed with Student’s *t*-test. Data were analyzed for significance among more than two groups by one-way ANOVA with Dunnett’s multiple comparison post-test. An associated probability (*p*) of ≤ 0.05 was considered significant.

### Target prediction and enrichment analysis

Putative targets of crizotinib, ceritinib, and capmatinib were identified based on structural similarity using the SMILES ID as an input in SwissTargetPrediction: http://www.swisstargetprediction.ch/ (26). Gene expression of the common targets was analyzed in CCA tumors when compared with normal bile duct from The Cancer Genome Atlas (TCGA) and plotted using the web tool, GEPIAv2 (http://gepia2.cancer-pku.cn/). Pathway enrichment for the target genes was performed using Enrichr(https://amp.pharm.mssm.edu/Enrichr/) and KEGG database (27–30).

## Results

### Differential endogenous expression of ALK, cMet in cholangiocarcinoma cell lines, and patient tumor samples

**Figure 1:**
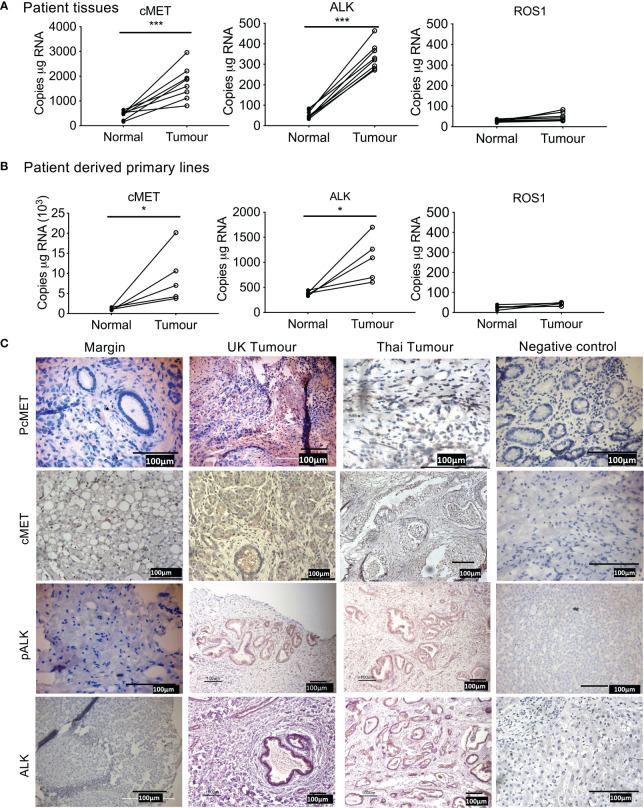
Expression of tyrosine kinase receptors in cholangiocarcinoma (CCA). Absolute mRNA expression (copies per µg of RNA) of cMET, ALK, and ROS1 was quantified using Droplet Digital PCR in surgically resected CCA patient tumor samples of eight UK patients **(A)**, and four primary lines derived from surgically resected tissue **(B)** compared to tissue resected margins. Immunohistochemistry staining for phosphorylated cMET (pcMET), cMET, phosphorylated ALK (pALK), and ALK protein levels in CCA tissues **(C)**. Scale bars = 100 µM. Significant difference was determined by paired *t*-test. *p < 0.005, **p < 0.01, ***p < 0.001 compared to margin. Negative controls (IgG).

We first determined the expression of cMet, ALK, and ROS1 in patient tumor tissue compared to tissue resection margin. mRNA expression of Met and ALK was significantly elevated in patient tissue (**Figure 1A**) and tumor cells isolated from primary tissue (**Figure 1B**) compared to their respective normal margins; however, ROS1 expression was barely detectable in both tumor samples and resected margins.

Immunohistochemistry of tumor tissue sections from patients both from Thailand (presumed OV positive) and from the UK (presumed OV negative) were positive for phosphorylated cMet, cMet receptor, phosphorylated ALK, and ALK receptor with similar intensities (**Table 1**^1^, **Figure 1C**) compared with histologically normal tissues taken from resection margin of CCA. cMet and ALK receptors and activated pcMet and pALK receptors had expression that was more intense in tumor cells from CCA patients irrespective of their global origin.

**Table 1:** Levels of staining in cholangiocarcinoma patient tissue samples.

	pcMET	cMET	pALK	ALK
No staining	0/15	0/15	0/15	0/15
Weak	0/15	0/15	0/15	0/15
Moderate	5/15 (33%)	2/15 (13.3%)	1/15 (6%)	0/15(0%)
Strong	10/15 (66%)	13/15 (86%)	14/15 (93%)	15/15 (100%)

^1^Table 1.0: Values are represented as actual and percentages in pcMET; phosphorylated mesenchymal-epithelial transition factor, MET; mesenchymal-epithelial transition factor, pALK; phosphorylated Anaplastic Lymphoma Kinase, ALK Anaplastic Lymphoma Kinase.

**Figure 2:**
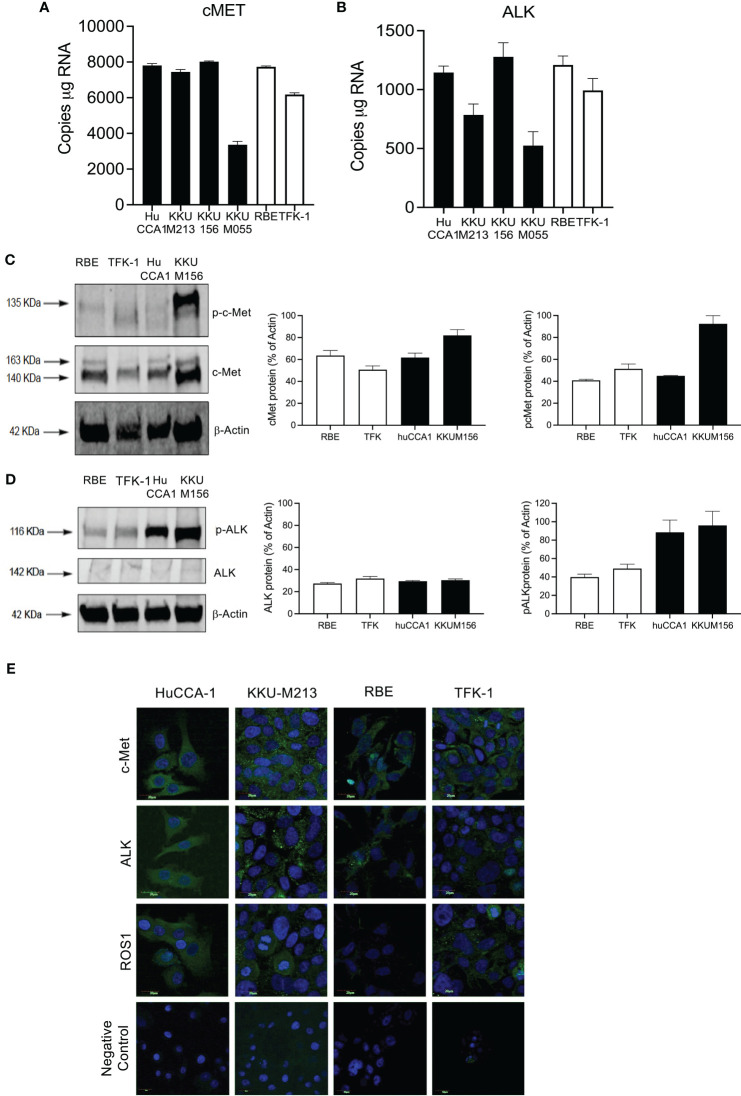
Expression of cMET, ALK, and ROS1 in the cholangiocarcinoma cell line. Absolute expression of cMET **(A)** and ALK **(B)** was determined in six immortalized cell lines: derived from *Opisthorchis viverrinae* (OV)-infected patients: HuCCA1, KKU-M213, KKU-M156, and KKU-M055 (solid bars) and two Non-Ov lines (open bars); RBE and TFK-1. Protein expression of C-MET and p-cMET **(C)** and ALK and p-ALK **(D)** was determined by Western blot. Full Western blot images are in **Supplementary Figure 3A**. Protein localization of c-Met, ALK, and ROS1 was observed in CCA cell lines with specific primary antibodies followed by probing secondary antibodies conjugated with Alexa488 and counterstained with DAPI **(E)**.

We next confirmed that the high expression of cMet and ALK and low expression of ROS1 was preserved in immortalized CCA cell lines (**Figure 2**). cMet and ALK expression was investigated in six CCA cell lines; four cell lines were derived from patients with OV infection, namely, KKU-M055, KKU-M213, KKU-M156, and HuCCA-1, and two lines from OV-negative patients, RBE and TFK-1. Transcript expression of all cell lines showed significant cMet and ALK levels (**Figures 2A, B**), respectively. cMet and ALK protein was expressed in all cell lines. Activated forms of cMet and ALK receptors were increased in OV-induced CCA cell lines compared to non-OV-induced CCA cell lines, but this was not correlated with transcript expression levels (**Figures 2C, D**, respectively). The protein and transcript expression of ROS1 in CCA cell was not detected by ddPCR or Western blot (data not shown). Immunofluorescence staining confirmed ALK and cMet expression in cell lines, but barely detectable levels of ROS1 were shown in all cell lines (**Figure 2E**).

### Receptor activation status in CCA

**Figure 3:**
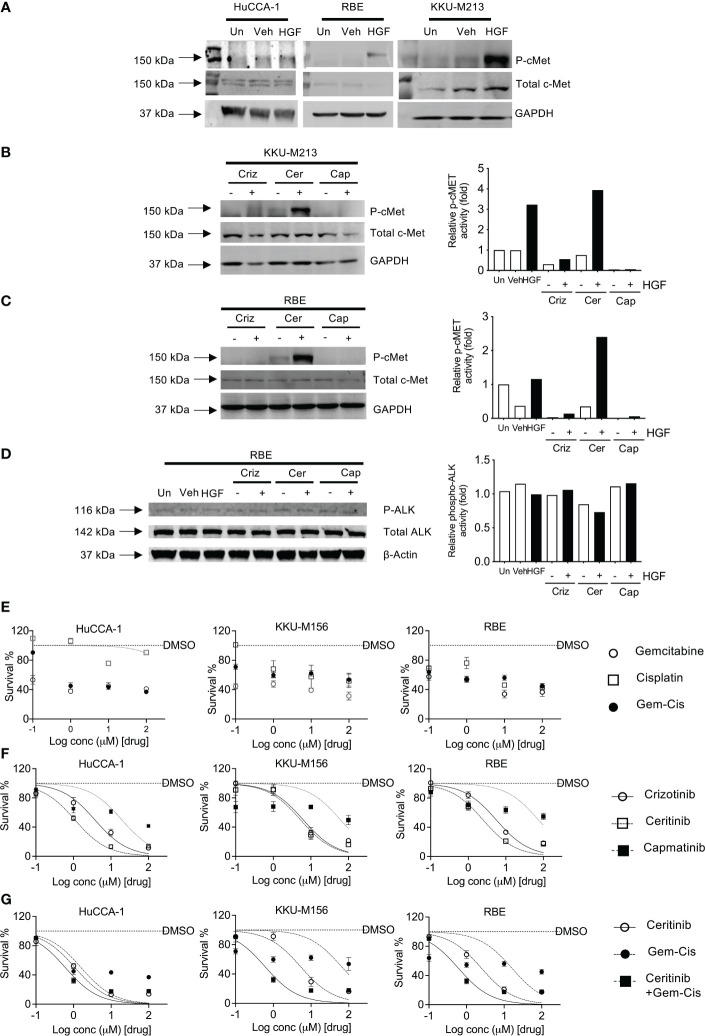
Activation and inhibition of cMET receptor in CCA cell lines. cMET receptor activation was induced by HGF-1 in HuCCA-1, RBE, and KKU-213 **(A)** compared with untreated (Un) and vehicle-treated (Veh) controls. Full Western blot images are in **Supplementary Figure 3B**. Inhibition of HGF-induced receptor activation of cMET in KKU-213 **(B)** and RBE **(C, D)**. Full Western blot images are in **Supplementary Figure 3C** top and bottom panel. The percentage of cell survival of CCA cell lines treated with increasing doses of gemcitabine and cisplatin combinations **(E)**, crizotinib, ceritinib, capmatinib and combinations of gemcitabine- and cisplatin-treated CCA cell lines **(F)**, ceritinib co-incubations with gemcitabine and cisplatin treatment in CCA cell lines **(G)**. Lines are dose–response curve fits. No lines are shown when data could not be fitted.

The CCLE database that has fusion data for multiple cancer lines including CCA lines show that KKU-213 and RBE do not present with ALK fusions. Hence, we sought to explore the HGF/cMet axis. We determined receptor stimulation status, and this was achieved by stimulating with HGF, a cMet ligand. Post-stimulation, Western blot analysis was employed to determine the activity of the receptor through phosphorylation. All cell lines were able to be stimulated with HGF ligand, shown by an increase in the level of phosphorylated cMet receptor. However, the levels of activation were higher in OV-induced CCA cell lines compared to non-OV-induced ones (**Figure 3A**).

### Sensitivity of CCA cells to treatment with tyrosine kinase and chemotherapeutic inhibitors in activated CCA cell lines

Four CCA cell lines were employed to determine the sensitivity to crizotinib—a cMet, ALK, and ROS1 inhibitor, ceritinib—an ALK inhibitor, and capmatinib (INCB28060)—a cMet-specific inhibitor. Receptor activity was blocked using inhibitors prior to stimulation using HGF ligand. Crizotinib treatment reduced phosphorylated cMet in both unstimulated and stimulated cells without affecting total cMet, phosphorylated ALK, and ALK receptor levels (**Figures 3B, C**). Ceritinib treatment showed no change in p-cMet post-stimulation compared to unstimulated and when stimulated by HGF in both cells lines and had no effect on total cMet. Capmatinib treatment inhibited both untreated and treated phospho-cMet. ALK was not phosphorylated by HGF, and its baseline phosphorylation was not affected by crizotinib, ceritinib, or capmatinib (**Figure 3D**).

We then investigated the effects of crizotinib, ceritinib, capmatinib, and gemcitabine–cisplatin combinations in five CCA cell lines. Gemcitabine and cisplatin as single treatments or in combination showed little effect on cell growth (**Figure 3E**; **Supplementary Figure 2A**) with IC_50_ ranging from 16 to 150 μM (**Supplementary Figure 2B**, **S2** **Table 2**^2^). Ceritinib was cytotoxic, in a dose-dependent manner (IC_50_ ranging from 1 to 9 μM), as was crizotinib (IC_50_ 3.7–11.6 μM) whereas capmatinib was not particularly cytotoxic (IC_50_ 19.51–246 μM, **Figure 3F**). To determine whether ceritinib inhibitors in addition to existing therapies would be more advantageous, we examined the effect of ceritinib in the presence of current chemotherapeutic agents gemcitabine and cisplatin combinations (**Figure 3G**). We first confirmed the sensitivity of the cells to ceritinib to be more sensitive than gemcitabine and cisplatin treatment (ceritinib IC_50_ 1–9 μM, gemcitabine and cisplatin IC_50_ 16–148.9 μM). Combinations of ceritinib and gemcitabine and cisplatin proved to be even more cytotoxic in a dose-dependent manner (IC_50_ 0.60–2.32 μM) (**Figure 3G**; **S2** **Table 2**.0^2^) with CI value towards synergism (**Supplementary Figure 2C**). These results indicate that the growth inhibitory effects of gemcitabine and cisplatin can be enhanced by the addition of ceritinib in CCA.

**Table 2:** Sensitivity of cholangiocarcinoma cell lines to treatment.

Cell line	Gem-Cis (µM)	Crizotinib (µM)	Ceritinib (µM)	Capmatinib (µM)	Crizotinib +Gem-Cis (µM)	Ceritinib + Gem-Cis (µM)
RBE	16.08	5.572	2.485	74.29	21.18	0.767
TFK-1	148.9	12.79	1.691	246	19.24	0.9544
HuCCA-1	1.59	3.711	1.09	19.51	1.545	0.6041
KKU-M055	103.5	11.59	9.029	140.7	33.52	2.247
KKU-M156	64.27	6.25	5.288	61.15	32.95	0.6535

^2^Table 2.0: IC_50_ values of all cell lines present in μM.

### Sensitivity of inhibitors in primary CCA cells

**Figure 4:**
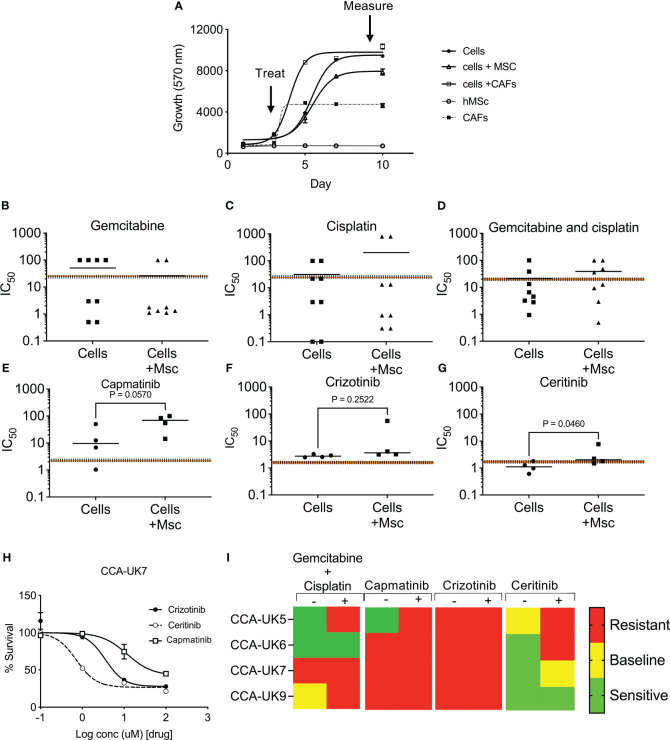
Sensitivity of inhibitors in primary cell derived from cholangiocarcinoma patients in 3D tumor growth assays (TGA). Four primary cell lines were derived from surgically resected CCA tissue samples: CCA-UK5, CCA-UK6, CCA-UK7, and CCA-UK9 **(A)**. Sensitivity of primary cells to gemcitabine **(B)**, cisplatin **(C)**, and combinations of gemcitabine and cisplatin **(D)** on four primary cell lines. Sensitivity of primary cells to capmatinib **(E)**, crizotinib **(F)**, and ceritinib **(G)** with and without co-culturing with humanized mesenchymal cells (+Mscs). Paired *t*-tests. Sensitivity of primary cells to crizotinib, ceritinib, and capmatinib **(H)**. All cell survival growth was measured by AlamarBlue. The mean ± 20% peak serum concentration for patients is given as the dotted and shaded lines. Heatmap of mean IC_50_ relative to mPSC levels **(I)**.

We then assessed the response of crizotinib, ceritinib, and capmatinib in four primary CCA cell cultures isolated from patients, in a 3D tumor growth assay, in the presence of human mesenchymal cells (MSCs). Cells were grown in 3D and treated, and cell survival was measured (**Figures 4A, B**). While all four patient-derived cell cultures tested showed some sensitivity to gemcitabine and cisplatin treatment that was at the same level or below the mean peak serum concentration (mPSC) for patients undergoing treatment with these agents [intravenous infusion over 30 min gemcitabine (1,000 mg/m^2^) and 2 h for cisplatin (25 mg/m^2^)], the addition of MSCs increased the resistance to the combined treatments and in four cases above the mPSC, indicating that while cells from these two tumors appear to be sensitive without mesenchymal support, they would be insensitive to treatment clinically (**Figures 4B–D**).

Cells were insensitive to capmatinib and crizotinib, with higher IC_50_s than the mPSC, and addition of MSCs made them even less sensitive (i.e., more resistant) (**Figures 4E, F**). In contrast, all four primary cells were sensitive (IC_50_ less than mPSC) to ceritinib treatment and borderline sensitive when co-cultured with MSCs (**Figure 4G**), suggesting the potential for some clinical response. An example of one primary cell culture is given in **Figure 4H**, and a heatmap of sensitivity is provided in **Figure 4I**. These results show that the majority of the primary CCA cells are sensitive to ceritinib, even in a more tumor-relevant setting.

### Mechanistic insight into the role of ceritinib treatment in CCA

To determine the possible mechanism of action for ceritinib treatment, we measured the levels of apoptosis and induction of autophagy post manipulation of ALK, cMet, and ROS receptor.

**Figure 5:**
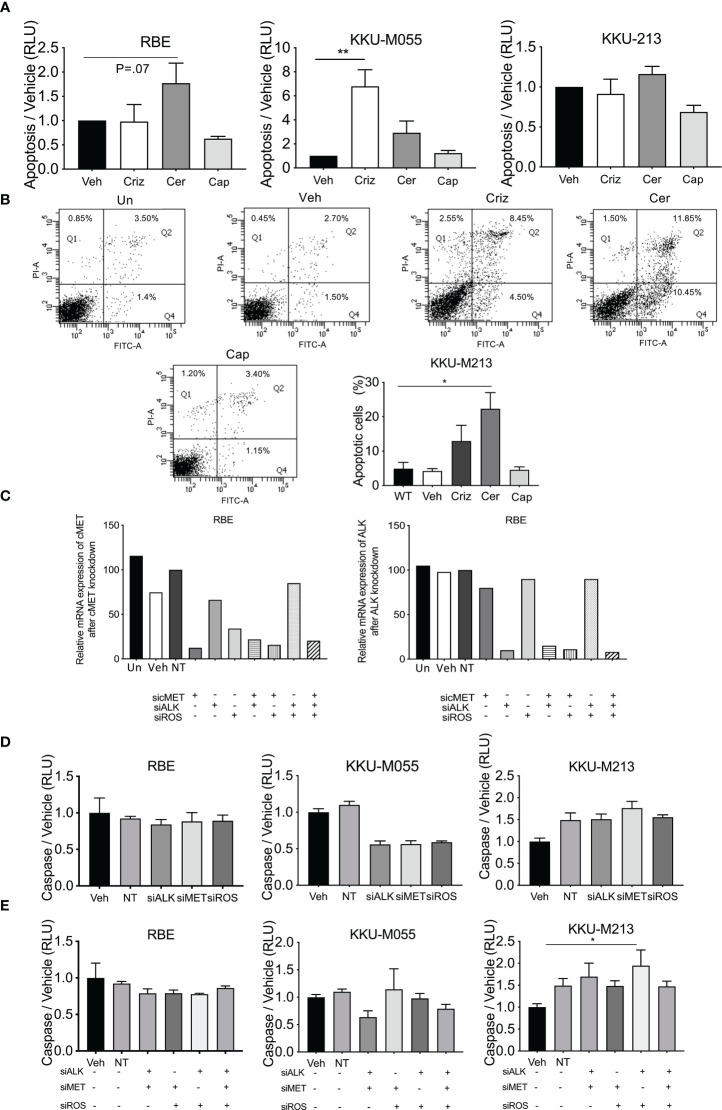
Levels of apoptosis post-drug treatment and receptor inhibition. Apoptosis was determined in crizotinib-, ceritinib-, and capmatinib-treated cells by cleave caspase 3/7 glo **(A)**. Apoptotic cells were determined by Annexin V and PI in untreated control, vehicle, crizotinib, ceritinib, and capmatinib and the percentage of apoptotic cells were measured **(B)**. Absolute expression of ALK and MET post-siRNA knockdown of cMET, ALK, and ROS1 **(C)**. Caspase activity of single knockdown **(D)** and of multiple siRNA knockdowns as demonstrated in the panel **(E)**. Data were measured for significance by one-way ANOVA with Dunnett’s multiple comparison post-test. *p < 0.05, **p < 0.01, and ***p < 0.001 compared to vehicle and NT controls.

Apoptosis was determined by measuring caspase 3/7 using caspase Glo assay (**Figure 5A**) and Annexin V staining (**Figure 4B**) in five cell lines treated with crizotinib, ceritinib, and capmatinib. Increased caspase activity was observed in one cell line (KKU-M055) post crizotinib, and was increased in RBE cells post-ceritinib treatment relative to vehicle-treated cells, and no caspase difference was observed in capmatinib-treated cells relative to vehicle-treated cells (**Figures 5A, B**; **Supplementary Figure 3A**).

The effect of apoptosis post-siRNA knockdown of ALK, Met, and ROS manipulations as single knockdowns was determined. ddPCR revealed that Non-Target and Sham treated cells had similar levels of expression of cMet and ALK (**Figure 5C**) to vehicle-treated cells. Transfected cells showed 90% knockdown for cMet and ALK in all cell lines by their respective siRNA. Combined cMet and ROS1 knockdown also induced a reduction in ALK expression (**Figure 5C**).

siRNA knockdown of cMet, ALK, and ROS1 did not increase caspase activity in any cell line, and reduced it in one cell line (**Figure 5D**; **Supplementary Figure 3B**). Combined knockdown of ALK and Met augmented a small increase in the levels of apoptosis. However, combined knockdown of cMet and ROS1 had no effect on the levels of apoptosis. Combined knockdown of ALK and ROS1 significantly increased levels of apoptosis in one cell line and so did triple knockdown (**Figure 5E**; **Supplementary Figure 3C**). These results suggest that apoptosis was not being induced as a general mechanism of cell death in the CCA lines.

**Figure 6:**
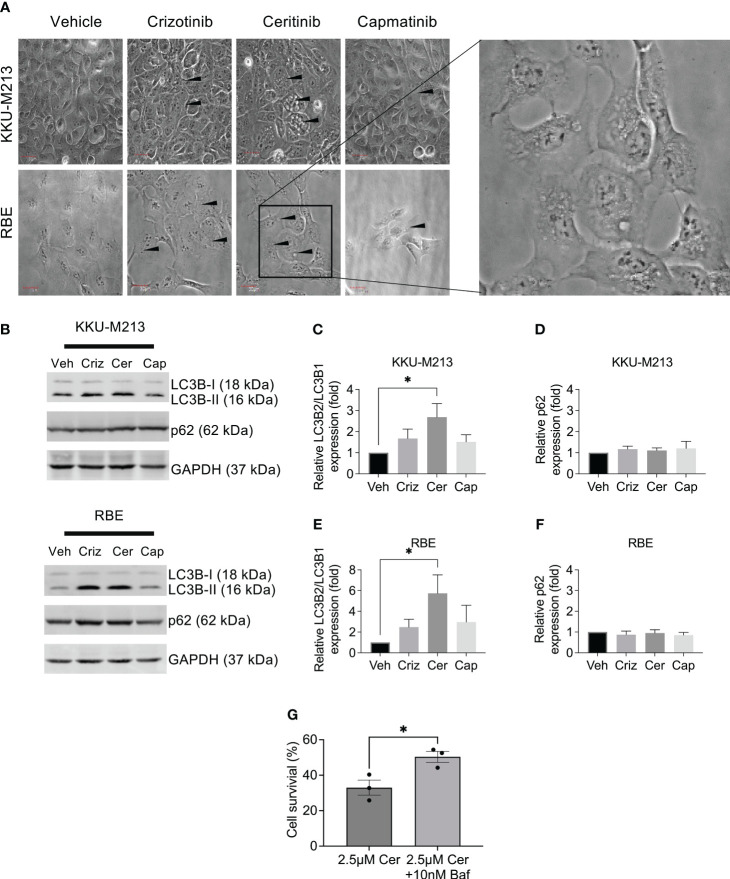
Autophagy induction by ceritinib treatment. Crizotinib-, ceritinib-, and capmatinib-treated KKU-M213 and RBE cells. Vacuolation, elongated cells with little cell–cell interaction demonstrated with black arrow **(A)**. Western blot analysis of autophagy markers in CCA cells lines KKU-M213 and RBE after treatment with crizotinib, ceritinib, and capmatinib **(B–F)**. KKU-M213 cell viability after ceritinib or ceritinib+bafilomycin treatment at 24 h **(G)**. Full Western blot images are in **Supplementary Figure 3D**. Data were measured for significance with one-way ANOVA with Dunnett’s multiple comparison post-test. *p < 0.05, **p < 0.01, and ***p < 0.001 relative to vehicle-treated cells.

To investigate alternative cell death pathways induced post-treatment with ceritinib, crizotinib, and capmatinib, using phase contrast microscopy, we observed ceritinib-induced changes in the cell morphology and while vehicle-treated control cells have the typical cuboidal cell morphology, ceritinib-treated cells lost their cuboidal shapes and cell–cell contact and developed an elongated shape with cytoplasmic extensions. Ceritinib treatment induced an accumulation of vacuoles and dilation of autolysosomes (**Figure 6A**), suggestive of a possible autophagy mechanism. The turnover of autophagosomal marker levels LC3B-1, II, and p62 was examined by immunoblotting post-treatment with ceritinib, crizotinib, and capmatinib. Ceritinib treatment induced the conversion of LC3B-I to its lapidated form, LC3B-II. LC3B-II accumulation was present in ceritinib-treated cells. Furthermore, p62 levels were unaffected (**Figures 6B–F**). These results suggest that autophagic activity could be a mechanism of cell death induced post-treatment with ceritinib. To test this, we determined whether the effect could be reversed using an autophagy flux specific inhibitor bafilomycin. KKU-M213 cells and RBE cells were pre-treated with bafilomycin and then subjected to ceritinib, crizotinib, and capmatinib treatments and cell survival was determined. Bafilomycin partially reversed the effect of ceritinib (**Figure 6G**). In contrast, bafilomycin enhanced the effect of crizotinib treatments in KKU-M213 and RBE cells and had no effect on capmatinib-treated cells (**Supplementary Figures 4A–C**).

### Predictive targets and pathways of ceritinib treatment

**Figure 7:**
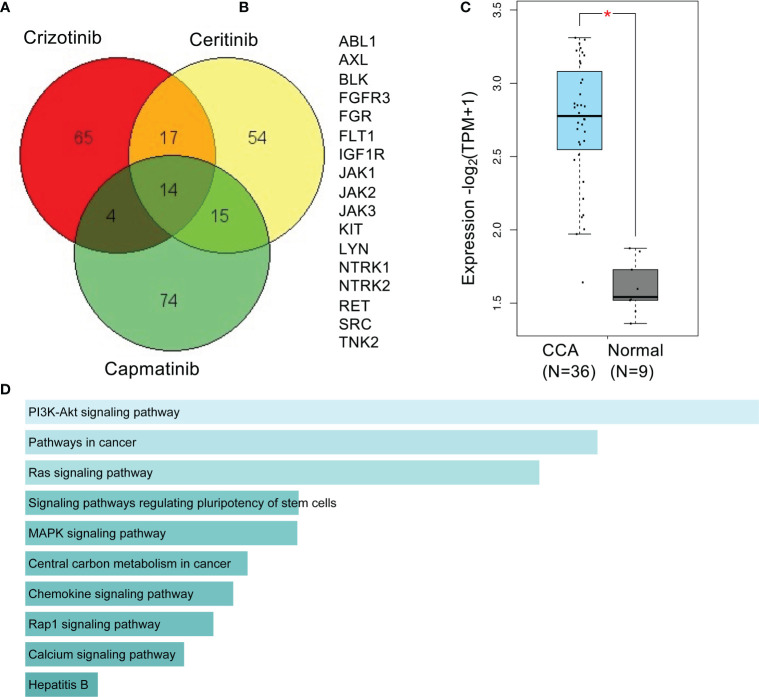
Predictive targets and pathways of crizotinib and ceritinib mechanism of action in CCA. Venn diagram of predicted targets of crizotinib, ceritinib, and capmatinib. Canonical SMILES structures of crizotinib, ceritinib, and capmatinib were acquired from PubChem database. Using the SMILES structures, target prediction was performed by SwissTargetPrediction by Swiss Institute of Bioinformatics **(A)**. A total of 17 common predicted targets of crizotinib and ceritinib excluding predicted targets of capmatinib **(B)**. Gene signature expression patterns of common 17 target genes in CCA (TCGA) database compared to normal bile duct, analyzed on GEPIA2.0. **(D)** KEGG_pathway enrichment of 17 target genes by Enrichr **(C, D)**. *P < 0.05.

A Venn diagram of predicted targets of crizotinib, ceritinib, and capmatinib reveals 17 targets common to crizotinib- and ceritinib-treated cells (**Figures 7A, B**). An increased gene signature of these 17 targets in TCGA databases was involved compared to normal bile duct (**Figure 7C**). High enrichment for 10 pathways were found especially for PI3K, pathways in cancer, and RAS signalling pathway (**Figure 7D**).

## Discussion

Treatment of bile duct cancer still has a very poor prognosis. Demonstration of a statistically significant increase in survival with gemcitabine and cisplatin was the first identification of a chemotherapeutic approach in patients, and recent studies have shown that addition of immunotherapy (durvalumab) can also enhance survival, but still 80% of patients succumb even on triple treatment. A number of targeted therapies have failed in patients including sorafenib (VEGFR and other tyrosine kinases) (31), erlotinib (EGFR inhibitor) (32), afatinib, (ERB2 antagonist) (33), and cabozantinib (VEGFR/MET inhibitor) (34), but the only effective targeted kinase inhibitors are those targeting FGFR in those small proportion of patients with FGFR fusions, translocation, or activating mutations (35, 36). Therefore, the need for targeted treatment in patients with CCA is still high, and identification of targets for kinase inhibitors is still required.

Our studies show that cMet and ALK and their active phosphorylated receptors (pcMet and pALK) are overexpressed in Thai and UK CCA tissue, UK primary cells, and different Thai CCA cell lines irrespective of their OV status. ROS1 expression was barely detectable, but high levels of ROS1 expression have been shown to be an indicator of better disease-free survival, indicating that the patient samples may be aggressive cholangiocarcinomas (37). However, CCA primary cell treatment with crizotinib or the cMet inhibitor capmatinib in 3D culture showed insensitivity (i.e., resistance) and the addition of humanized mesenchymal cells showed further insensitivity to crizotinib treatment. This insufficient effect of crizotinib suggests a potential lack of efficacy *in vivo*. The use of close-to-patient models (primary derived cells) in combination with humanized mesenchymal cells provides a more clinically relevant test of drug efficacy than using immortalized cells grown in 2D on plastic, which over time will have resulted in a drift from the parental original clone (38). It has been shown that cancer cells derived from patient tissue are more phenotypically and genetically related than immortalized cells (22) and their behavior in the presence of mesenchymal cells more closely reflects the tumor microenvironment (39).

To determine the causative mechanism of this action, we employed the cMet-specific inhibitor, capmatinib, and an ALK/ROS inhibitor, ceritinib. Capmatinib had no effect on cell survival in CCA cell lines or primary cells derived from CCA patient tissue. Capmatinib proved to be less potent than current chemotherapy regimens of gemcitabine and cisplatin treatments, had no effect on apoptosis, and was excluded for further progression within the study.

Surprisingly, ceritinib, which is an ALK inhibitor, did have a significant inhibitory effect on CCA cells. ALK is a highly conserved receptor tyrosine kinase belonging to the insulin receptor superfamily (40). Aberrant ALK expression has been documented in CCA, but the use of an ALK specific inhibitor has not been investigated.

We show that treatment with ceritinib dose-dependently inhibited growth and survival response of a panel of CCA cell lines more potently than crizotinib, capmatinib, and combinations of gemcitabine and cisplatin, without inhibiting cMet phosphorylation. The sensitivity to ceritinib was associated with the expression of ALK. We then determined the efficacy on growth inhibition of the combined treatment with gemcitabine, cisplatin, and ceritinib. We found an additive effect of ceritinib to the combinations of gemcitabine and cisplatin. We show that all primary cell lines were highly sensitive to ceritinib treatment in 3D culture with IC_50_ values lower than the mean peak serum concentration levels. Although co-culturing them with humanized mesenchymal cells increased the IC_50_ levels, they were still at or lower than the mean peak serum concentration levels, suggesting a more likelihood of translation through to positive response clinically. The results here suggest that the ALK inhibitor ceritinib is a promising treatment in CCA alone or in combinations with gemcitabine and cisplatin.

The mechanism of effect for ceritinib in CCA is unknown. Our studies show that treatment with ceritinib did not induce apoptosis whereas crizotinib induced apoptosis only in one cell line. Apoptosis levels were measured after single knockdown of cMet, ALK, and ROS1 receptors inhibiting the levels of apoptosis. Dual knockdown of receptors showed no increase in levels of apoptosis except for one cell line. Crizotinib is a multitarget inhibitor (41) and the knockdown of all three receptors showed no induction of apoptosis. This could be because crizotinib exerts its effects through the modulation of the growth, migration, and invasion of malignant cells.

However, following treatment with ceritinib, the cell morphology changed to elongated cells with fewer cell–cell interactions, higher recruitment of vacuoles in the cytoplasm, and dilation of autolysosomes, consistent with an autophagic mechanism (42). Ceritinib treatment also indicated a conversion of LC3B-I to its lipidated form, LC3B-II, compared to vehicle-treated without any further changes in p62 levels, suggesting that ceritinib exerts its effects through autophagy, and this treatment highlighted 17 downstream targets that could prove to be interesting downstream targets.

ALK receptors have been shown to be the primary driver of several cancers including anaplastic large cell lymphoma, lung cancer, and neuroblastoma as a consequence of fusion with oncogenes, gene amplification, and protein overexpression (10, 16, 43). Ceritinib is FDA-approved for NSCLC patients with ALK and ROS1 rearrangements. These rearrangements occur in 1% of patients and are rare. However, our studies show for the first time that ceritinib is a potent inhibitor for CCA irrespective of the rearrangement status, subtype specificity, and OV infection status. This could be due to its structurally distinct second-generation confirmation or that anaplastic lymphoma kinase could be the primary driver of cholangiocarcinoma disease and progression. Despite rapid and dramatic effects of crizotinib in other cancer types and its ineffectiveness on cholangiocarcinoma, a common concern of target therapy is the development of resistance, and in the case of NSCLC, resistance to crizotinib inevitably occurs with years of treatment. Crizotinib resistance occurs due to mutations of ALK and ROS (6). Resistance to ceritinib is certainly possible but has not yet been shown.

## Conclusions

In conclusion, we demonstrate for the first time the potential therapeutic benefits of ceritinib in combination with gemcitabine and cisplatin, which exert anti-tumor activity in cholangiocarcinoma.

## Data availability statement

The original contributions presented in the study are included in the article/**Supplementary Material**. Further inquiries can be directed to the corresponding authors.

## Ethics statement

The studies involving humans were approved by UK National Research Ethics Service. The studies were conducted in accordance with the local legislation and institutional requirements. The participants provided their written informed consent to participate in this study.

## Author contributions

KY-U, RT, KM, AG, and DB: concept and design. KY-U, KM, MS-C, PC, BB, and SV: methodology and data acquisition. KY-U, KM, MS-C, PC, BB, SV, and AG: analysis and interpretation of data (e.g., image analysis, statistical analysis etc.). KY-U, DB, AG, RT, and KM: writing, review and revision of the manuscript. KY-U, DB, and RT: study supervision. All authors contributed to the article and approved the submitted version.

## Supplementary material

Supplementary Figure 1 sensitivity if CCA cells lines to treatment of MET, ALK and ROS1 inhibitors. The percentage of cell survival of CCA cell lines treated with increasing doses of Gemcitabine and cisplatin combinations in KKU-M055 and TFK-1 cells **(A)**, Crizotinib, Ceritinib, capmatinib and combinations of Gemcitabine and Cisplatin treated KKU-M055, KKU-M213 and TFK-1 cell lines **(B)**. confidence interval measured for Ceritinib and gemcitabine and cisplatin co incubations **(C)**.

Supplementary Figure 2 levels of apoptosis post drug treatment and receptor inhibition. Apoptosis was determined by cleave caspase 3/7 glo in HuCCA-1 and TFK-1 cell lines **(A)**. Caspase activity of single knockdown **(B)** and of multiple siRNA knockdowns as demonstrated in the figure **(C)** in HuCCA1 and TFK-1 cell lines. Data was measured for significance by one-way ANOVA with Dunett’s multiple comparison post test. *p < 0.05, **p < 0.01 and ***p < 0.001 compared to vehicle and NT controls.

Supplementary Figure 3 supporting info: whole blots for western blot analysis. Full western blot analysis for expression for protein expression of C-MET and p-cMET and ALK and p-ALK **(A)**. Western blot analysis of inhibition of HGF-induced receptor activation of cMET in HuCCA1 **(B)** KKU-213 and RBE **(C)**. Western blot analysis of autophagy markers in KKU-M213 and RBE cell lines after treatment with crizotinib, ceritinib and capmatinib **(D)**.
