# Supplementary material for: Preclinical evidence for anaplastic lymphoma kinase inhibitors as novel therapeutic treatments for cholangiocarcinoma
Source: Front Oncol. 2023 Dec 7;13:1184900. doi: 10.3389/fonc.2023.1184900 (PMC10748508; doi:10.3389/fonc.2023.1184900)
Supplement: Supplementary file 1 [file DataSheet_1.pdf]

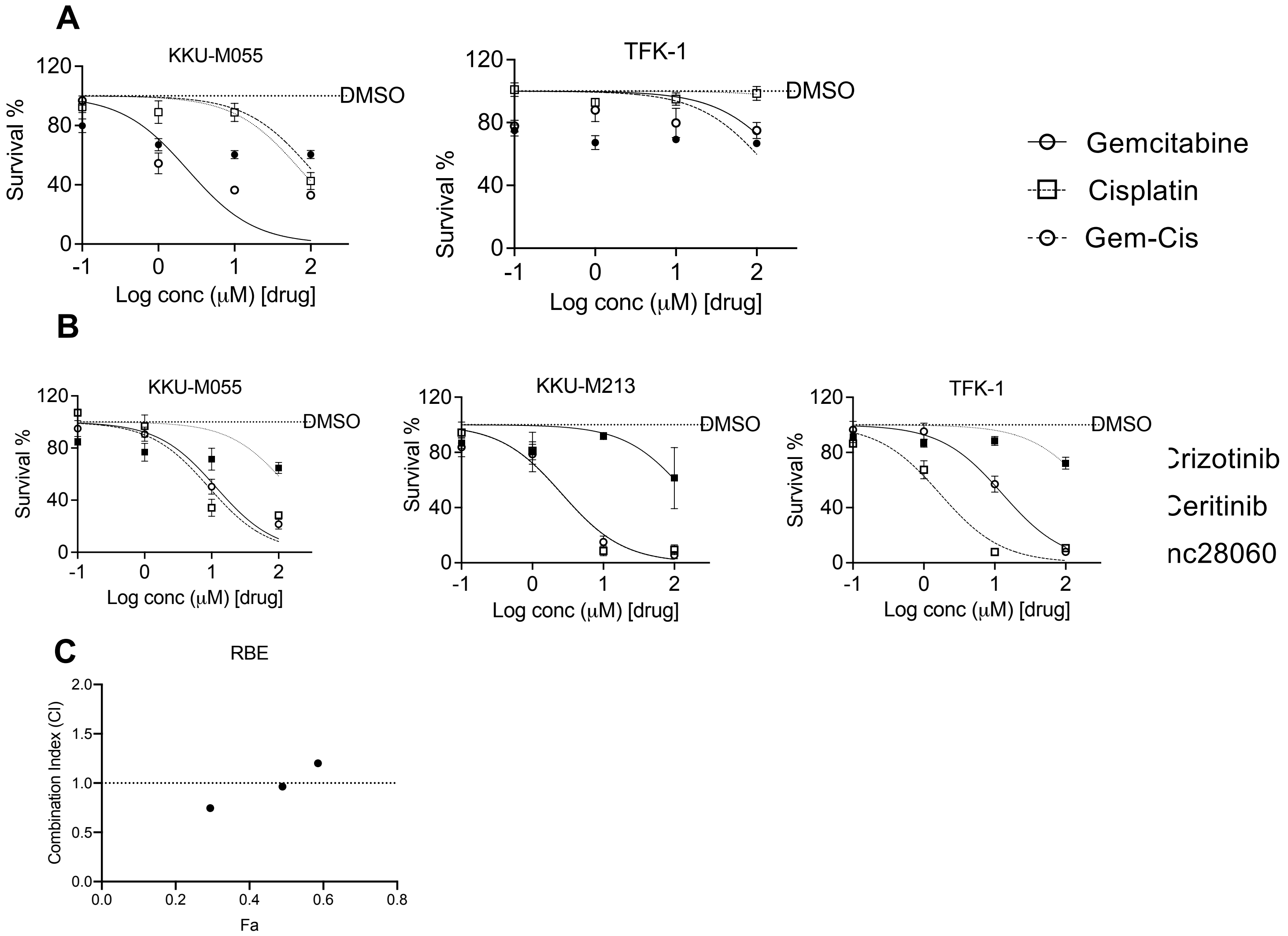

Table 2.0

Cell line	Gem-Cis (μM)	Crizotinib (μM)	Ceritinib (μM)	Inc28060 (μM)	Crizotinib +Gem-Cis (μM)	Ceritinib + Gem-Cis (μM)
RBE	16.08	5.572	2.485	74.29	21.18	0.767
TFK-1	148.9	12.79	1.691	246	19.24	0.9544
HuCCA-1	1.59	3.711	1.09	19.51	1.545	0.6041
KKU-M055	103.5	11.59	9.029	140.7	33.52	2.247
KKU-M156	64.27	6.25	5.288	61.15	32.95	0.6535
