# Supplementary figures and images for: Preclinical evidence for anaplastic lymphoma kinase inhibitors as novel therapeutic treatments for cholangiocarcinoma
Source: Front Oncol. 2023 Dec 7;13:1184900. doi: 10.3389/fonc.2023.1184900 (PMC10748508; doi:10.3389/fonc.2023.1184900)

**A**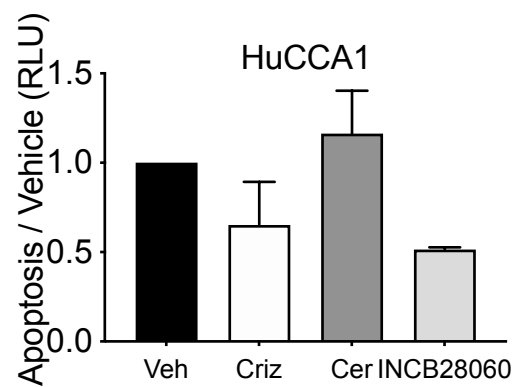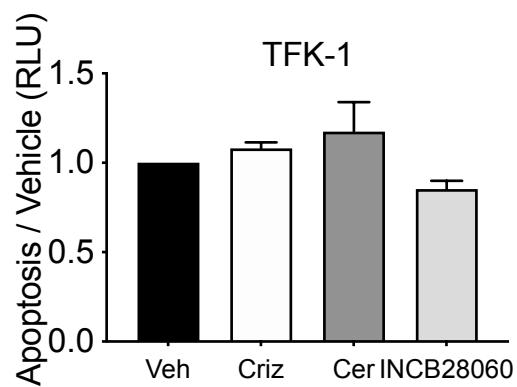**B**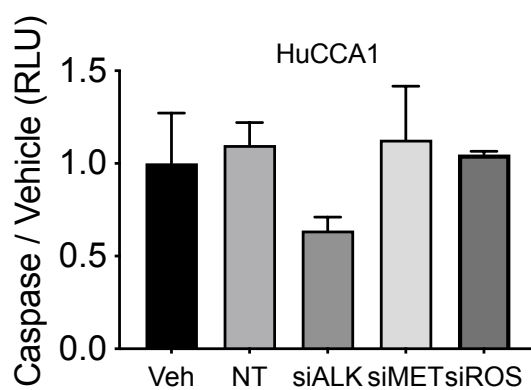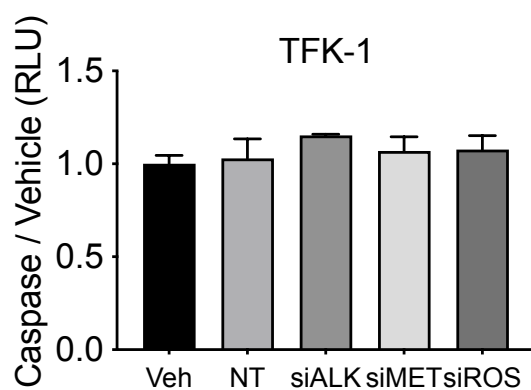**C**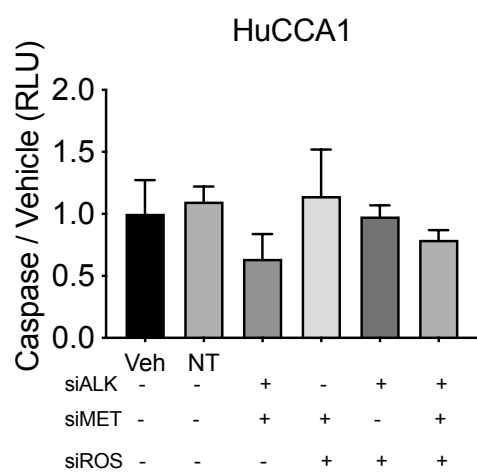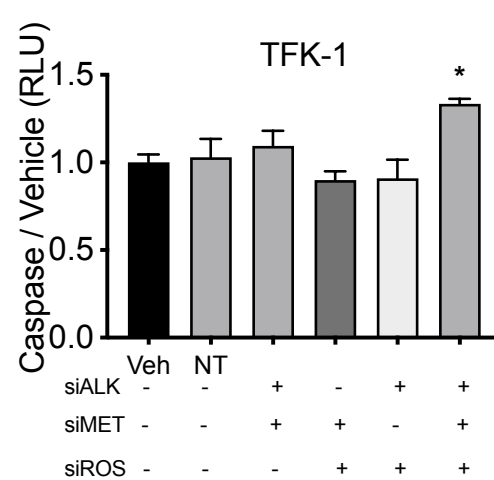

Supplement: Supplementary Figure 2 — levels of apoptosis post drug treatment and receptor inhibition. Apoptosis was determined by cleave caspase 3/7 glo in HuCCA-1 and TFK-1 cell lines (A). Caspase activity of single knockdown (B) and of multiple siRNA knockdowns as demonstrated in the figure (C) in HuCCA1 and TFK-1 cell lines. Data was measured for significance by one-way ANOVA with Dunett’s multiple comparison post test. *p < 0.05, **p < 0.01 and ***p < 0.001 compared to vehicle and NT controls. [file Image_2.pdf]

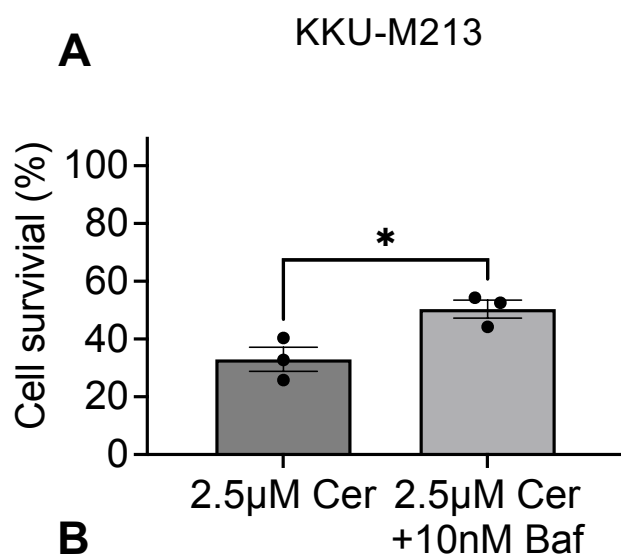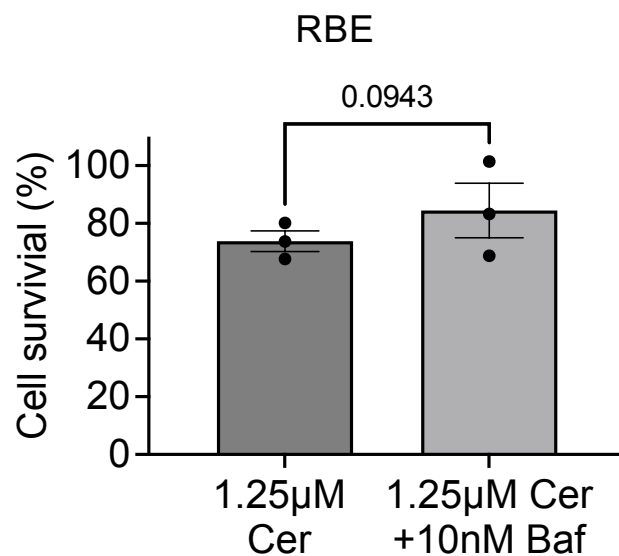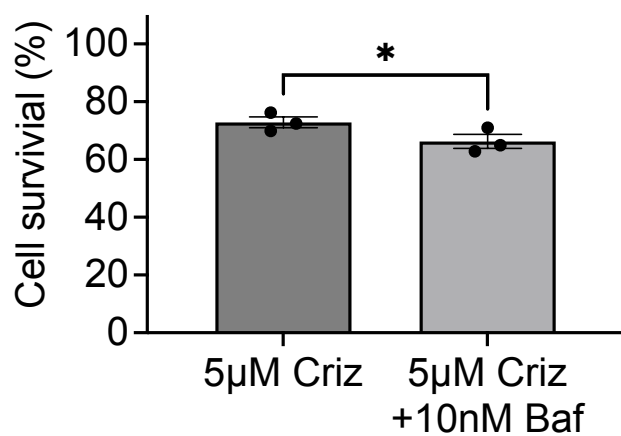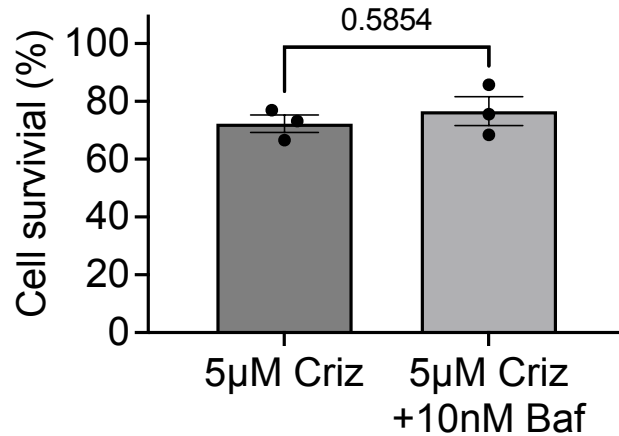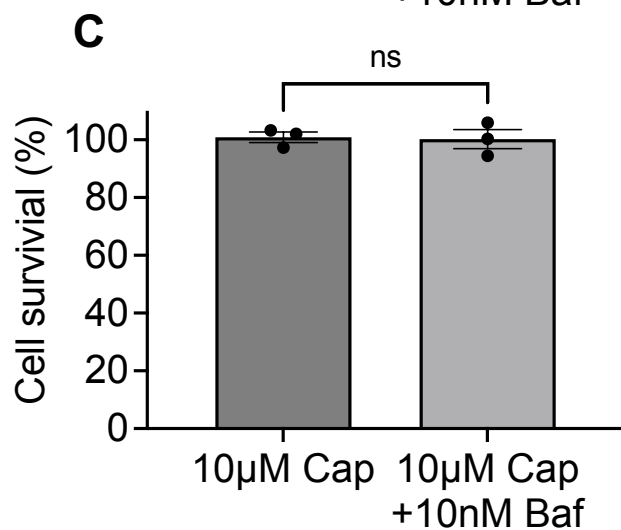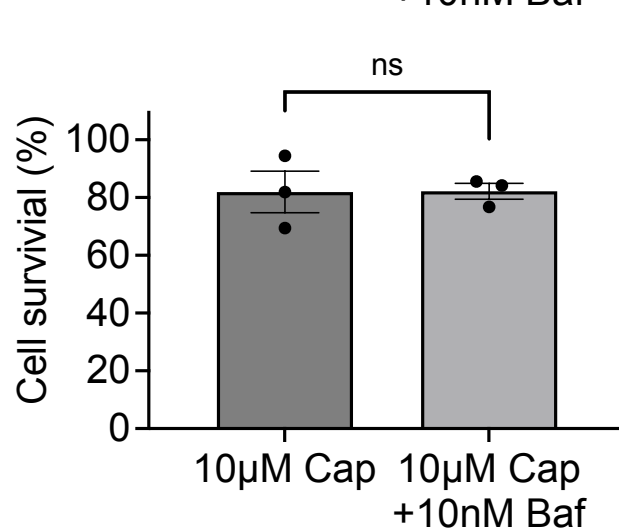

Supplement: Supplementary file 5 [file Image_4.pdf]
