# Supplementary material for: Preclinical evidence for anaplastic lymphoma kinase inhibitors as novel therapeutic treatments for cholangiocarcinoma
Source: Front Oncol. 2023 Dec 7;13:1184900. doi: 10.3389/fonc.2023.1184900 (PMC10748508; doi:10.3389/fonc.2023.1184900)
Supplement: Supplementary Figure 3 — supporting info: whole blots for western blot analysis. Full western blot analysis for expression for protein expression of C-MET and p-cMET and ALK and p-ALK (A). Western blot analysis of inhibition of HGF-induced receptor activation of cMET in HuCCA1 (B) KKU-213 and RBE (C). Western blot analysis of autophagy markers in KKU-M213 and RBE cell lines after treatment with crizotinib, ceritinib and capmatinib (D). [file Image_3.pdf]

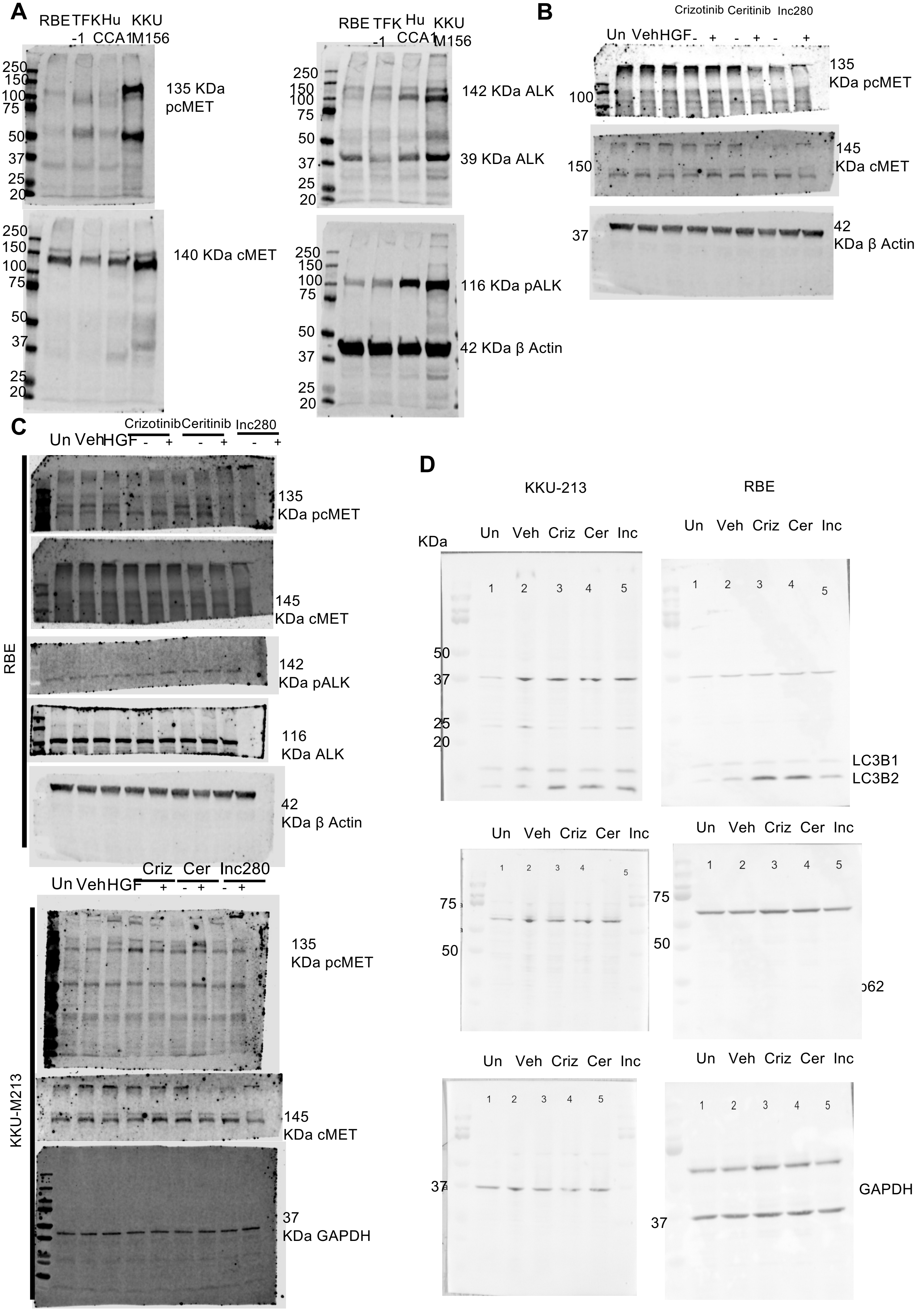
A
RBE TFK-1 Hu CCA1 KKU M156
250
150
100
75
50
37
25
20
135 KDa pcMET
140 KDa cMET
142 KDa ALK
39 KDa ALK
116 KDa pALK
42 KDa β Actin
B
Crizotinib Ceritinib Inc280
Un Veh HGF - + - + - +
135 KDa pcMET
145 KDa cMET
42 KDa β Actin
C
Crizotinib Ceritinib Inc280
Un Veh HGF - + - + - +
RBE
135 KDa pcMET
145 KDa cMET
142 KDa pALK
116 KDa ALK
42 KDa β Actin
Criz Cer Inc280
Un Veh HGF + - + - +
KKU-M213
135 KDa pcMET
145 KDa cMET
37 KDa GAPDH
D
KKU-213
RBE
KDa
Un Veh Criz Cer Inc
1 2 3 4 5
LC3B1
LC3B2
p62
GAPDH
